# Status of Legislation and Regulatory Control of Public Health Pesticides in Countries Endemic with or at Risk of Major Vector-Borne Diseases

**DOI:** 10.1289/ehp.1103637

**Published:** 2011-07-08

**Authors:** Graham Matthews, Morteza Zaim, Rajpal Singh Yadav, Agnes Soares, Jeffrey Hii, Birkinesh Ameneshewa, Abraham Mnzava, Aditya Prasad Dash, Mikhail Ejov, Soo Hian Tan, Henk van den Berg

**Affiliations:** 1 Imperial College, Ascot, United Kingdom; 2 Vector Ecology and Management, Department of Control of Neglected Tropical Diseases, World Health Organization, Geneva, Switzerland; 3 World Health Organization, Regional Office for the Americas, Washington, DC, USA; 4 World Health Organization, Regional Office for the Western Pacific, Manila, Philippines; 5 World Health Organization, Regional Office for Africa, Harare, Zimbabwe; 6 World Health Organization, Regional Office for the Eastern Mediterranean, Cairo, Egypt; 7 World Health Organization, Regional Office for South-East Asia, New Delhi, India; 8 World Health Organization, Regional Office for Europe, Copenhagen, Denmark; 9 Kuala Lumpur, Malaysia; 10 Laboratory of Entomology, Wageningen University, Wageningen, the Netherlands

**Keywords:** environment, health risks, malaria, pesticide legislation, pesticide management, pesticide regulation, public health pesticides, vector-borne diseases, vector control

## Abstract

Background: Legislation and regulation of pesticides used in public health are essential for reducing risks to human health and the environment.

Objective: We assessed the global situation on legislation and regulatory control of public health pesticides.

Methods: A peer-reviewed and field-tested questionnaire was distributed to 142 member states of the World Health Organization (WHO); 113 states completed the questionnaire.

Results: Legislation on public health pesticides was absent in 25% of the countries. Where present, legislation often lacked comprehensiveness, for example, on basic aspects such as labeling, storage, transport, and disposal of public health pesticides. Guidelines or essential requirements for the process of pesticide registration were lacking in many countries. The capacity to enforce regulations was considered to be weak across WHO regions. Half of all countries lacked pesticide quality control laboratories, and two-thirds reported high concern over quality of products on the market. National statistics on production and trade of pesticides and poisoning incidents were lacking in many countries. Despite the shortcomings, WHO recommendations were considered to constitute a supportive or sole basis in national registration. Also, some regions showed high participation of countries in regional schemes to harmonize pesticide registration requirements.

Conclusions: Critical deficiencies are evident in the legislative and regulatory framework for public health pesticides across regions, posing risks to human health and the environment. Recent experience in some countries with situational analysis, needs assessment, action planning, and regional collaboration has signaled a promising way forward.

Major vector-borne diseases such as malaria, lymphatic filariasis, dengue, leishmaniasis, Chagas disease, and Japanese encephalitis, as well as nuisance pests in and around human habitations, cause tremendous suffering and impede the alleviation of poverty and constrain economic development. Thus, attaining the United Nation’s Millennium Development Goals (United Nations 2011) is difficult. Recent reports indicate considerable progress in reducing malaria and neglected tropical diseases [World Health Organization (WHO) 2010c, 2010f]. Vector control using pesticides has remained an important component in combating these diseases, and the use of pesticides in many countries has been increasing with the scaling up of interventions. A global assessment of trends in public health pesticide use is forthcoming.

If not properly regulated, the use of vector-control pesticides and other public health pesticides, which include those for use by households and pest control operators (WHO 2010d), could undermine the effectiveness of interventions or pose risks to human health and the environment. The WHO recommends only a limited number of pesticides for public health purposes (WHO 2006a), excluding those that are known to be most hazardous to human health and the environment. Under the *International Code of Conduct on the Distribution and Use of Pesticides* [Food and Agriculture Organization of the United Nations (FAO) 2005], hereafter referred to as the Code of Conduct, and two recent World Health Assembly (WHA) resolutions, WHA 63.25 and 63.26 (WHO 2010e), countries and parties are urged to establish or strengthen capacity for the regulation of the sound management of pesticides, which include agricultural and public health pesticides, throughout their life cycle. Also, several legally binding international instruments, to which any country can be a party, are in place to ensure sound management of pesticides. The main instruments are the Stockholm Convention on Persistent Organic Pollutants (2011), the Basel Convention on the Control of Transboundary Movements of Hazardous Waste and Their Disposal (2011), and the Rotterdam Convention on the Prior Informed Consent Procedure for Certain Hazardous Chemicals and Pesticides in International Trade (2011).

There are indications that regulations for vector-control pesticides and other public health pesticides are inadequate in many countries at risk of vector-borne diseases, as shown in a preliminary study conducted in 2003 (WHO 2004). To verify these critical findings, a new study with expanded scope and increased coverage was undertaken in 2010. The study’s objective was to map the global landscape on the management of public health pesticides in countries endemic with or at risk of major vector-borne diseases. This would provide a baseline to assist in developing strategies to strengthen the capacity for pesticide management in WHO member states.

Here we report on the outcome of part of the questionnaire, relating to legislation and regulatory control of public health pesticides during their life cycle (Figure 1). Regulatory control involves pesticide registration and the enforcement of legislation. Registration is the process of evaluation and approval by central authorities to determine which pesticide products are permitted to be used and for what purposes and to exercise control over aspects such as quality, use levels, and labeling of pesticides, thus ensuring that the interests of end users and the environment are protected (WHO/FAO 2010a). Collectively, legislation, registration, and enforcement are the three instruments through which central authorities can exercise significant control over how pesticides should be managed. In another report, we presented the second part of the questionnaire, relating to pesticide management in the practice of vector control (van den Berg et al. 2011).

**Figure 1:**
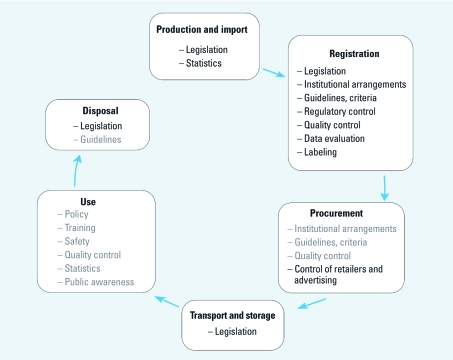
Stages of the life cycle of vector-control pesticide products, from production and import to waste disposal, with aspects of pesticide management pertaining to each stage. Boldface text indicates management aspects addressed in this article; gray text indicates aspects addressed in a separate report (van den Berg et al. 2011).

## Materials and Methods

A questionnaire was used to collect data from the WHO member states. The questionnaire had been developed through a WHO-consultative process, field tested in selected countries, and peer reviewed before being finalized (WHO 2011b). The questionnaire was translated into three languages; the English version was administered with most countries, except where the French or Spanish version was preferred. Questionnaires were provided to the six WHO regional offices in Africa (Harare), the Americas (Washington, DC), the eastern Mediterranean (Cairo), Europe (Copenhagen), Southeast Asia (New Delhi), and the western Pacific (Manila). These offices distributed the questionnaires to WHO representatives’ offices in the 142 WHO member states (territories excluded)that are endemic with or at risk of one or more of the major diseases transmitted by insect vectors mentioned above: malaria, lymphatic filariasis, dengue, leishmaniasis, Chagas disease, and Japanese encephalitis. The study excluded Australia, all but six of the European countries (Azerbaijan, Georgia, Kyrgyzstan, Tajikistan, Turkey, Uzbekistan), Japan, New Zealand, Canada, and the United States.

The part of the questionnaire addressed in this article was completed by national pesticide registration authorities, through the facilitation of the WHO country offices. Most of the questions gave choices between two options; some had more options. The data were entered into a computer spreadsheet for analysis. Missing records and records with more than one option selected were excluded from analysis. The questionnaire did not allow for comprehensive assessment of individual countries, and we did not attempt quantitative comparison between countries or regions. In the analysis, large countries were counted equally as small ones.

## Results

Of the 142 targeted countries representing a total population of 5.4 billion in six WHO regions, 113 countries responded to the questionnaire, representing 94% of the population in all targeted countries [see Supplemental Material, Table 1 (http://dx.doi.org/10.1289/ehp.1103637)]. This is a response rate of 80% of the number of targeted countries (Table 1). Not all countries responded to all questions in the questionnaire. The response rate was lowest in the African region, which was mainly attributable to logistic issues in some countries. We used the aggregated responses of the questionnaire, presented in Table 2, to draw attention to key issues in three major themes, legislation, registration, and enforcement, with data stratified by region. For topics having similar results across regions, we refer to the aggregated results in Table 2.

**Table 1:** Number of countries and their populations that were targeted and responded to the questionnaire in six WHO regions.

No. of countries					Population (× 10^6^)				
WHO region	Targeted	Responded	Percent	Targeted	Responded	Percent
Africa		46		30		65		805		614		76
Americas		33		28		85		570		549		96
Eastern Mediterranean		21		17		81		580		568		98
Europe		6		5		83		126		99		78
Southeast Asia		11		8		73		1,760		1,686		96
Western Pacific		25		25		100		1,639		1,639		100
All		142		113		80		5,481		5,155		94


**Table 2:** Questions as formulated in the first part of the questionnaire, with the percentage of countries giving a positive response to each question.

		
Values in parentheses are number of positive responses/number of countries that responded to each question.		

*Legislation.* Overall, 84% of countries reported having national or regional legislation for registration and control of pesticides, but in the African and Southeast Asian regions, a quarter of countries reported the absence of such legislation (Table 3). In most countries where pesticide legislation did exist, this included public health pesticides, but in the eastern Mediterranean region public health pesticides were included in the legislation in only 77% of countries (Table 3).

**Table 3:** Status of pesticide legislation in the WHO regions: presence of national and/or regional legislation for registration and control of pesticides, and whether it covers the registration and control of public health pesticides.

WHO region	Presence of legislation	Legislation covers public health pesticides
Africa		73 (22/30)		82 (18/22)
Americas		89 (24/27)		91 (21/23)
Eastern Mediterranean		87 (13/15)		77 (10/13)
Europe		100 (5/5)		100 (5/5)
Southeast Asia		75 (6/8)		100 (6/6)
Western Pacific		91 (20/22)		95 (18/19)
All		84 (90/107)		89 (78/88)
Data are the percentage of countries responding positively to each question in each region. Values in parentheses are number of positive responses/number of countries that responded to each question.				

Legislation should be comprehensive, covering aspects of labeling, storage, transport, and disposal of public health pesticides, to promote proper use and reduce risks to human health and the environment throughout a pesticide’s life cycle (FAO 2005). Labeling of containers is essential to conveying information on use, safety, and proper disposal to end users. Yet, only 72% of countries reported having legislation for labeling; figures were lowest in the African and eastern Mediterranean regions (Table 4). Moreover, legislation for safe storage, safe transport, and proper waste disposal was in place in 72%, 63%, and 56% of all countries, respectively (Table 4). The African and eastern Mediterranean regions scored lowest in most categories of legislation. Transport of pesticides is an important matter; vehicles should be specially adapted for safe transportation, and measures should be available to avoid spillage and prevent environmental pollution (WHO 2003). Pesticide-related waste should not be mixed with municipal waste, where it could contaminate the environment.

**Table 4:** Presence of national legislation for container labeling, safe storage, safe transport, and proper waste disposal of public health pesticides in the WHO regions.

WHO region	Labeling	Safe storage	Safe transport	Proper disposal
Africa		63 (19/30)		60 (18/30)		47 (14/30)		53 (16/30)
Americas		76 (19/25)		75 (18/24)		63 (15/24)		38 (9/24)
Eastern Mediterranean		50 (8/16)		63 (10/16)		56 (9/16)		50 (8/16)
Europe		100 (5/5)		80 (4/5)		100 (5/5)		80 (4/5)
Southeast Asia		75 (6/8)		86 (6/7)		75 (6/8)		75 (6/8)
Western Pacific		88 (21/24)		87 (20/23)		78 (18/23)		72 (18/25)
All		72 (78/108)		72 (76/105)		63 (67/106)		56 (61/108)
Data are the percentage of countries responding positively to each question in each region. Values in parentheses are number of positive responses/number of countries that responded to each question.								

*Registration.* One single national authority for registration of all pesticides is promoted under the Code of Conduct (WHO/FAO 2010a). In 71% of countries responding, vector-control pesticides and agricultural pesticides were registered by the same authority, which in most cases was housed within the ministry of agriculture. However, in the eastern Mediterranean region, most countries had separate authorities (Table 5). It is noteworthy that pesticides applied directly to humans and household pesticide products were more commonly registered under separate authorities.

**Table 5:** Status of pesticide registration in the WHO regions: vector-control pesticides and agricultural pesticides registered by one authority, published national guidelines available for registration of public health pesticides, WHOPES recommendations required for registration of public health pesticides, and where WHOPES recommendations are required, they are accepted as the sole basis for national registration.

WHO region	One registration authority	Registration guidelines	Require WHOPES recommendations	WHOPES as sole basis
Africa		82 (14/17)		45 (13/29)		79 (23/29)		48 (11/23)
Americas		71 (12/17)		73 (19/26)		60 (15/25)		13 (2/15)
Eastern Mediterranean		42 (5/12)		67 (10/15)		80 (12/15)		42 (5/12)
Europe		60 (3/5)		100 (5/5)		80 (4/5)		25 (1/4)
Southeast Asia		80 (4/5)		86 (6/7)		71 (5/7)		0 (0/5)
Western Pacific		79 (15/19)		50 (12/24)		80 (16/20)		19 (3/16)
All		71 (53/75)		61 (65/106)		74 (75/101)		29 (22/75)
Data are the percentage of countries responding positively to each question in each region. Values in parentheses are number of positive responses/number of countries that responded to each question.								

Published national guidelines for the registration of public health pesticides, needed to help ensure that registration requirements are met and transparency is promoted, were reported from only 61% of countries and were least common in the African and western Pacific regions (Table 5).

Recommendations by the WHO Pesticide Evaluation Scheme (WHOPES; WHO 2011d) were required for registration of public health pesticides in 74% of countries (Table 5). In most of these countries, WHOPES recommendations served as a supportive element in pesticide registration. However, in the African and eastern Mediterranean regions, WHOPES recommendations served as the sole basis for registration in almost half of the countries.

Regional pesticide registration schemes have potential advantages of work sharing and harmonization (Organization for Economic Cooperation and Development 2010). Almost half of all countries reported being part of a regional scheme, and in the African region this was 77% (Table 6). No such collaboration was reported from the Southeast Asian region.

**Table 6:** Status of registration of public health pesticides in the WHO regions: participation in a regional pesticide registration scheme, registration in the country of origin required to apply for registration of public health pesticides, locally generated data required to support registration, and legislation provisions for reregistration and periodic review of public health pesticide products.

WHO region	Regional participation	Registration in country of origin	Require locally generated data	Require reregistration
Africa		77 (23/30)		63 (19/30)		79 (23/29)		66 (19/29)
Americas		58 (15/26)		73 (19/26)		48 (12/25)		84 (21/25)
Eastern Mediterranean		40 (6/15)		81 (13/16)		46 (6/13)		60 (9/15)
Europe		20 (1/5)		100 (5/5)		40 (2/5)		100 (5/5)
Southeast Asia		0 (0/7)		57 (4/7)		71 (5/7)		63 (5/8)
Western Pacific		25 (6/24)		70 (16/23)		45 (10/22)		76 (16/21)
All		48 (51/107)		71 (76/107)		57 (58/101)		73 (75/103)
Data are the percentage of countries responding positively to each question in each region. Values in parentheses are number of positive responses/number of countries that responded to each question.								

More than 70% of countries reported the prerequisite that a product must be registered in its country of origin, as part of their application dossier for registration of new public health pesticides (Table 6). The figure ranged from 57% in Southeast Asia to 100% in the five responding European countries. Moreover, most countries regarded the registration in other countries as supportive for the national registration, as shown in Table 2.

Strikingly, only 57% of countries required local data in support of registration of a public health pesticide to determine local effectiveness and suitability. This requirement was common in the African and Southeast Asian regions (Table 6).

Reregistration and periodic review of pesticides is promoted under the Code of Conduct, requiring new data to be collected and appropriate regulatory action to be taken (WHO/FAO 2010a). Seventy-three percent of countries reported having a provision in their legislation to periodically review and reregister individual pesticides (Table 6).

*Enforcement.* National pesticide regulations in the health sector were reported to be enforced “to a large extent” in only 41% of countries, as shown in Table 2, indicating an acknowledged gap in regulatory enforcement. The reported level of enforcement was lowest in the African (33%) and western Pacific (27%) regions and in the five participating countries in the European region (data not shown).

One of the possible consequences of inadequately enforced regulations is the presence of substandard and counterfeit pesticides on the market, which could lead to ineffective and inefficient use, pesticide resistance in vectors, and increased risks for human health and the environment (WHO/FAO 2011). There was concern in all regions about substandard or counterfeit public health pesticides, with 67% of all countries reporting it to be “a major/moderate problem” (Table 2). This was particularly important in the African (83%) and American and European regions (data not shown).

Half of the countries reported having a national pesticide quality control laboratory. The western Pacific and African regions had the fewest countries with such laboratories (Table 7). Only 19 of 54 countries (35%) that did not have quality control laboratories reported seeking the assistance of foreign laboratories to check for the quality of their products. It is expected the remaining 35 countries could not enforce quality control.

**Table 7:** Status of regulatory enforcement of public health pesticides in the WHO regions: national pesticide quality control laboratory present, pest control operators required to be licensed or certified, and regulations in place to control advertisement of pesticides and prevent reuse of pesticide containers by the public.

WHO region	Presence of quality control laboratory	Certification of pest control operators	Control of advertisements	Preventing reuse of containers
Africa		40 (12/30)		77 (23/30)		67 (20/30)		41 (12/29)
Americas		52 (14/27)		68 (17/25)		36 (9/25)		44 (11/25)
Eastern Mediterranean		81 (13/16)		56 (9/16)		38 (6/16)		50 (8/16)
Europe		80 (4/5)		60 (3/5)		20 (1/5)		80 (4/5)
Southeast Asia		86 (6/7)		75 (6/8)		38 (3/8)		63 (5/8)
Western Pacific		25 (6/24)		73 (16/22)		58 (14/24)		58 (14/24)
All		50 (55/109)		70 (74/106)		49 (53/108)		50 (54/107)
Data are the percentage of countries responding positively to each question in each region. Values in parentheses are number of positive responses/number of countries that responded to each question.								

It is essential that the pesticide applicators are skilled and competent, especially where human dwellings have to be treated with pesticides (WHO 2006b). Overall, 70% of countries reported that pest control operators were required to be licensed or certified, which implies that many countries lack this regulatory control, particularly in the eastern Mediterranean region (Table 7).

Regulatory control over advertisement, needed to help ensure the correct and appropriate communication of information in marketing of public health pesticides (WHO/FAO 2010b), was reportedly in place in less than half of all countries, with substantial differences between regions (Table 7).

Unauthorized use of pesticides can result in ineffective application, unacceptable exposure, or residues in food. Yet, only two-thirds of countries indicated that they had a mechanism to prevent unauthorized use of pesticides (Table 2), with fairly similar results across regions. Also, although reuse of pesticide containers is a health hazard, by misusing the containers for keeping water or food, just 50% of countries reported having any regulation preventing reuse of pesticide containers by the public (Table 7).

Regarding local pesticide production, only 71% of the responding countries that had domestic manufacturing or formulation companies reported having regulations and certification for these companies to ensure that pesticide products meet national quality requirements (Table 2).

Regarding pesticide sales, 80% of countries reported having regulations to control retailers of agricultural pesticides, whereas 65% of countries had regulations to control retailers of public health pesticides (Table 2).

The importance of data collection for effective pesticide management has been stressed in the Code of Conduct (FAO 2005). Yet, only 78%, 49%, and 48% of responding countries reported having statistics available on import, local production, and export of pesticides, respectively, indicating important gaps in data collection in most regions (Table 8). Moreover, the Code of Conduct calls upon countries to investigate and document poisoning cases, but in our results, the regulatory authorities in only 39% of countries reported having access to aggregated data on pesticide poisoning (Table 2). Despite a paucity of published data on the number of cases, it is clear that human poisoning by pesticides, including public health pesticides, causes substantial morbidity and mortality, especially in developing countries (Jeyaratnam 1990; Kishi 2005).

**Table 8:** Availability of national statistics on imported pesticide products, locally produced or formulated public health pesticides, and export of pesticides, if applicable.

WHO region	Statistics on import	Statistics on local production	Statistics on export
Africa		72 (21/29)		38 (6/16)		31 (4/13)
Americas		86 (24/27)		47 (8/17)		60 (9/15)
Eastern Mediterranean		81 (13/16)		70 (7/10)		56 (5/9)
Europe		60 (3/5)		25 (1/4)		33 (1/3)
Southeast Asia		75 (6/8)		71 (5/7)		80 (4/5)
Western Pacific		76 (19/25)		43 (3/7)		29 (2/7)
All		78 (86/110)		49 (30/61)		48 (25/52)
Data are the percentage of countries responding positively to each question in each region. Values in parentheses are number of positive responses/number of countries that responded to each question.						

## Discussion

Comprehensive legislation and regulatory control that cover the life cycle of a pesticide are essential to safeguard the effective use of pesticides where they are needed and to reduce the risks they pose. Our study raises concern in areas of legislation, regulation, and enforcement of public health pesticides in countries endemic with or at risk of vector-borne diseases.

Pesticide legislation is currently absent from 16% of countries. Where legislation is present, it may not cover public health pesticides or their complete life cycle, and it may not meet current standards. Legislation was often lacking for basic aspects such as labeling, storage, transport, and disposal of public health pesticides.

Pesticide registration is in many countries constrained by a lack of published guidelines and a lack of essential registration requirements, such as the requirement to generate local data or to review and reregister a pesticide periodically. Consequently, hazardous or poor-quality pesticides may remain available for use for inappropriate purposes. Generation of local data is especially important for responding to the development of insecticide resistance in vector populations (WHO 2011c). Moreover, almost 30% of countries have a separate registration authority for vector-control pesticides and agricultural pesticides; hence, there is a prospect for merging the registration for all pesticides under one single authority. This would make more efficient use of resources and help avoid inconsistencies in the registration process (WHO/FAO 2010a).

The capacity to enforce public health pesticide regulations is weak across regions. This was evident from the lack of licensing of pest control operators, regulation of advertisement, and prevention of reuse of empty pesticide containers by the public, amid a lack of available statistics on pesticide imports, production, and export and pesticide poisoning cases. Because the number of countries that manufacture, formulate, or repackage pesticides is increasing, governments must ensure that national requirements are updated and enforced. Another pressing problem needing international attention is that half of the countries lack pesticide quality control facilities, and only one-third of those have been seeking assistance from foreign laboratories to test the quality of their pesticide products. Yet, substandard and counterfeit pesticides are a concern in most countries.

Hence, critical deficiencies in the legislative and regulatory framework of public health pesticides are evident in many countries endemic with or at risk of vector-borne diseases. Economic, sociocultural, and political drivers and externalities play a role as determinants of the current situation. However, where low priority is given to capacity building in pesticide management, this can probably be attributed more directly to a lack of awareness among policy makers and their advisers about the risks of pesticides (WHO 2010a). More in-depth study is needed to assess the situation in individual countries.

Against the observed shortcomings in pesticide management, and possibly because of them, WHOPES recommendations have played an important role: they were reportedly required by 74% of countries for the registration of public health pesticides. Specifically, in almost half of the countries in the African and eastern Mediterranean regions, WHOPES recommendations were used as the sole requirement for registration of public health pesticides. This demand for international standards and external guidance, in itself advantageous, emphasizes the responsibility of the WHO in supporting its member states to improve their management of public health pesticides (van den Berg et al. 2011).

Another positive observation is that some regions have shown high participation of countries in regional registration schemes, with potential to harmonize pesticide registration requirements and with potential to make efficient use of limited resources and expertise in pesticide evaluations through work sharing. The type and the quality of these schemes have not been assessed, but the African region showed the highest participation in regional registration schemes, an example of which is the Central Africa Inter-State Pesticides Committee (2011).

To improve the current situation, countries endemic with or at risk of vector-borne diseases need to strengthen their legislative and regulatory framework and associated specialist skills for pesticide management (WHO 2010a). This would require the collaboration and support of the ministry of health, the national pesticide regulatory authority, ministries of agriculture and environment, and municipalities. The stakeholders need to share their resources and expertise to best manage pesticides with their available resources. This would involve engaging scientists in the various agencies in data evaluation for registration and enforcement officers for carrying out regulatory activities. Countries also need financially sustainable mechanisms for monitoring the quality of pesticides, either through developing their own laboratory facilities or by enhancing cooperation with other countries. In the interim, countries could use the services of regional laboratories where available.

As part of an ongoing WHO project in the regions, a number of countries have recently conducted a situation analysis and needs assessment, on the basis of which they developed action plans on capacity building to strengthen public health pesticide management (Matthews et al. 2010; WHO 2010a, 2010b). Initial results indicate that the analytic and problem-solving methods used, involving various stakeholders and conducted within the context of an integrated vector management approach (WHO 2011a), are appropriate for raising the visibility of pesticide management on the national agenda and are beginning to address the complexities of pesticide management at all levels.

## Conclusions

Critical deficiencies are evident in the legislative and regulatory frameworks for public health pesticides among WHO member states and regions. This situation undermines the effective use of public health pesticides and poses unnecessary risks to human health and the environment in countries with vector-borne diseases.

Public health pesticide management requires political commitment, policy support, and adequate national and international resources and capacity to effectively deal with the issues at stake. This would involve awareness raising, information exchange, work sharing, and collaboration. In this regard, resolutions WHA 63.25 and 63.26 have reaffirmed a global commitment to pesticide management and emphasized the mandate of the WHO to facilitate implementation of appropriate strategies (WHO 2010e).

Recent experience in some countries with situation analysis, needs assessment, action planning, and regional collaboration has signaled a promising way forward.

## Supplemental Material

(84 KB) PDF
