# Supplementary material for: Status of Legislation and Regulatory Control of Public Health Pesticides in Countries Endemic with or at Risk of Major Vector-Borne Diseases
Source: Environ Health Perspect. 2011 Jul 8;119(11):1517–22. doi: 10.1289/ehp.1103637 (PMC3226504; doi:10.1289/ehp.1103637)
Supplement: (84 KB) PDF [file ehp.1103637.s001.pdf]

Supplemental Material

Status of Legislation and Regulatory Control of Public Health Pesticides in Countries Endemic with or at Risk of Major Vector-borne Diseases

Graham Matthews[1], Morteza Zaim[2], Rajpal Singh Yadav[2], Agnes Soares[3], Jeffrey Hii[4], Birkinesh Ameneshewa[5], Abraham Mnzava[6], Aditya Prasad Dash[7], Mikhail Ejov[8], Soo Hian Tan[9], and Henk van den Berg[10]

[1] Imperial College, Ascot, UK

[2] Vector Ecology and Management, Department of Control of Neglected Tropical Diseases, World Health Organization, Geneva, Switzerland

[3] World Health Organization, Regional Office for the Americas, Washington DC, USA

[4] World Health Organization, Regional Office for the Western Pacific, Manila, Philippines

[5] World Health Organization, Regional Office for Africa, Harare, Zimbabwe

[6] World Health Organization, Regional Office for the Eastern Mediterranean, Cairo, Egypt

[7] World Health Organization, Regional Office for South-East Asia, New Delhi, India

[8] World Health Organization, Regional Office for Europe, Copenhagen, Denmark

[9] 21 Lorong Abang Openg Lima, Taman Tun Dr Ismail, 60000 Kuala Lumpur, Malaysia

[10] Laboratory of Entomology, Wageningen University, Wageningen, the Netherlands

Address correspondence to:

Dr R.S. Yadav, Vector Ecology and Management, Department of Control of Neglected Tropical Diseases, World Health Organization, 20 Avenue Appia, 1211 Geneva 27, Switzerland. Telephone: +41 22 791 2961. Fax: +41 22 791 3111. Email: yadavraj@who.int

Table 1. Countries that responded to Part I and II of the questionnaire. Population size is indicated.

WHO Region	Country	Population[a]	Part I	Part II[b]
African	Benin	8 662 000	+	+
	Botswana	1 921 000	+	+
	Cameroon	19 088 000	+	+
	Chad	10 914 000	+	+
	Comoros	661 000	+	+
	Congo	3 615 000	+	+
	Côte d'Ivoire	20 591 000	+	+
	Democratic Republic of the Congo	64 257 000	+	+
	Eritrea	4 927 000	+	+
	Ethiopia	80 713 000	+	+
	Gambia	1 660 000	+	+
	Ghana	23 351 000	+	+
	Guinea	9 833 000	+	+
	Kenya	38 765 000	+	+
	Lesotho	2 049 000	+	+
	Liberia	3 793 000	+	+
	Madagascar	19 111 000	+	+
	Malawi	14 486 000	+	+
	Mauritania	3 215 000	+	+
	Mauritius	1 280 000	+	+
	Namibia	2 130 000	+	+
	Nigeria	151 212 000	+	+
	Rwanda	9 721 000	+	+
	Senegal	12 211 000	+	+
	Sierra Leone	5 560 000	+	+
	Swaziland	1 168 000	+	+
	Uganda	31 657 000	+	+
	United Republic of Tanzania	42 484 000	+	+
	Zambia	12 620 000	+	+
	Zimbabwe	12 463 000	+	+
Americas	Argentina	39 883 000	+	–
	Bahamas	338 000	+	+
	Belize	301 000	+	+
	Bolivia (Plurinational State of)	9 694 000	+	+
	Brazil	191 972 000	+	+
	Chile	16 804 000	+	+
	Colombia	45 012 000	+	+
	Costa Rica	4 510 000	+	+
	Dominica	67 000	+	+
	Dominican Republic	9 953 000	+	+
	Ecuador	13 481 000	+	+

Table 1 (cont.)

WHO Region	Country	Population[a]	Part I	Part II[b]
Americas (cont.)	El Salvador	6 134 000	+	+
	Grenada	104 000	+	+
	Guatemala	13 686 000	+	+
	Guyana	763 000	+	+
	Honduras	7 319 000	+	+
	Jamaica	2 708 000	+	+
	Mexico	108 555 000	+	+
	Nicaragua	5 667 000	+	+
	Panama	3 399 000	+	+
	Paraguay	6 238 000	+	+
	Peru	28 837 000	+	+
	Saint Kitts and Nevis	51 000	+	+
	Saint Lucia	170 000	–	+
	Suriname	515 000	+	+
	Trinidad and Tobago	1 333 000	+	+
	Uruguay	3 349 000	+	+
	Venezuela (Bolivarian Republic of)	28 121 000	+	+
Eastern Mediterranean	Afghanistan	27 208 000	+	+
	Bahrain	776 000	–	+
	Egypt	81 527 000	+	+
	Iran (Islamic Republic of)	73 312 000	+	+
	Iraq	30 096 000	+	+
	Jordan	6 136 000	+	+
	Libyan Arab Jamahiriya	6 294 000	+	+
	Morocco	31 606 000	+	+
	Oman	2 785 000	+	+
	Pakistan	176 952 000	+	+
	Qatar	1 281 000	+	+
	Saudi Arabia	25 201 000	+	+
	Somalia	8 926 000	+	+
	Sudan	41 348 000	+	+
	Syrian Arab Republic	21 227 000	+	+
	Tunisia	10 169 000	+	+
	Yemen	22 917 000	+	+
European	Azerbaijan	8 731 000	+	+
	Georgia	4 307 000	+	+
	Kyrgyzstan	5 414 000	+	+
	Tajikistan	6 836 000	+	+
	Turkey	73 914 000	+	+

Table 1 (cont.)

WHO Region	Country	Population[a]	Part I	Part II[b]
South-East Asia	Bangladesh	160 000 000	+	+
	Democratic Republic of Timor-Leste	1 098 000	+	+
	India	1 181 412 000	+	+
	Indonesia	227 345 000	+	+
	Maldives	305 000	+	+
	Nepal	28 810 000	+	+
	Sri Lanka	20 061 000	+	+
	Thailand	67 386 000	+	+
Western Pacific	Brunei Darussalam	392 000	+	+
	Cambodia	14 562 000	+	+
	China	1 344 920 000	+	+
	Cook Islands	20 000	+	+
	Fiji	844 000	+	+
	Kiribati	97 000	+	+
	Lao People's Democratic Republic	6 205 000	+	+
	Malaysia	27 014 000	+	+
	Marshall Islands	61 000	+	+
	Micronesia (Federated States of)	110 000	+	+
	Mongolia	2 641 000	+	+
	Nauru	10 000	+	+
	New Zealand	4 230 000	+	+
	Niue	2 000	+	+
	Palau	20 000	+	+
	Papua New Guinea	6 577 000	+	+
	Philippines	90 348 000	+	+
	Republic of Korea	48 152 000	+	+
	Samoa	179 000	+	–
	Singapore	4 615 000	+	+
	Solomon Islands	511 000	+	+
	Tonga	104 000	+	+
	Tuvalu	10 000	+	+
	Vanuatu	234 000	+	+
	Viet Nam	87 096 000	+	+
Grand total	113 countries		111	111

[a] WHO (2010)
[b] Results on Part II have been reported elsewhere (van den Berg et al. 2011)

References

van den Berg H, Hii J, Soares A, Mnzava A, Ameneshewa B, Dash AP, et al. 2011. Status of pesticide management in the practice of vector control: a global survey in countries at risk of malaria or other major vector-borne diseases. Malar J 10:125.

WHO (World Health Organization). 2010. World Health Statistics 2010. Geneva: World Health Organization. Available: http://www.who.int/whosis/whostat/2010/en/index.html [Accessed 29 June 2011]
